# Access to nutrition services and information after active cancer treatment: a mixed methods study

**DOI:** 10.1007/s11764-023-01352-x

**Published:** 2023-02-23

**Authors:** Brenton J. Baguley, Sarah Benna-Doyle, Shani Drake, Annie Curtis, Jane Stewart, Jenelle Loeliger

**Affiliations:** 1 Institute for Physical Activity and Nutrition, Deakin University, VIC 3220 Geelong, Australia; 2 School of Exercise and Nutrition Sciences, Deakin University, 221 Burwood Highway, VIC 3125 Burwood, Australia; 3 Nutrition and Speech Pathology Department, Peter MacCallum Cancer Centre, Melbourne, VIC 3000 Australia

**Keywords:** Nutrition, Cancer survivorship

## Abstract

**Purpose:**

Evidence-based guidelines for cancer strongly support nutrition and dietetic services for people with cancer and carers in order to improve patient-centred and health service outcomes. Access to nutrition services and information after completing active cancer treatment is relatively unknown in Australia. This study aimed to determine the availability, accessibility, barriers, and preferences to nutrition services and information after cancer treatment in Australia.

**Methods:**

Utilising mixed methods, people with cancer and carers completed a cross-sectional survey, and a sub-group of participants completed a semi-structured interview. The survey evaluated the availability of nutrition services, nutrition information searched, barriers, and preferences for nutrition information. Semi-structured interviews explored participant experience with nutrition services and information.

**Results:**

The 149 participants (including 10 carers) were predominately male and with a diagnosis of prostate cancer (63%). Overall, 23% of participants received nutrition information from a dietitian after cancer treatment. Participants (78%) indicated that accessing a nutrition specialist is the main barrier to receiving nutrition care after treatment. Most searched nutrition information on the internet (55%) and found the information easy to understand (89%), but conflicting (52%). Thematic analysis of interviews in fourteen cancer patients revealed three key themes pertaining to (1) preferred referral and timing of nutrition services, (2) lack of confidence in publicly available nutrition information, and (3) streamlining nutrition services for greater access.

**Conclusion:**

Access to a dietitian and evidence-based information after cancer treatment is limited for people with cancer and carers in Australia, despite the high interest and need for ongoing nutrition care.

**Implications for Cancer Survivors:**

Models of care evaluating the provision of appropriate nutrition care and information provision after cancer treatment are needed to address this unmet survivorship need.

**Supplementary Information:**

The online version contains supplementary material available at 10.1007/s11764-023-01352-x.

## Introduction

Australia has one of the highest cancer survival rates in the world [1, 2] due to advances in earlier detection and treatments in many cancers [3]. In Australia, an estimated 1.9 million people will be treated for cancer by 2040 [3], and therefore, the quality of life for people after cancer treatment is a high priority for national survivorship guidelines [4]. Up to 40% of people with cancer are diagnosed with malnutrition, with certain cancers associated with a higher risk of malnutrition (i.e. lung, head and neck, and gastrointestinal) due to the tumour location and side effects of treatment [5, 6]. International evidence-based guidelines highlight that people with cancer-related malnutrition experience reduced treatment compliance, survival, and quality of life during and after treatment [7]. In addition, people treated for breast or prostate cancer often experience weight gain, losses in muscle mass, and an increased risk of cardiometabolic side effects (i.e. cardiovascular disease and type II diabetes) [8–10]. Furthermore, dietary and exercise guidelines strongly recommend that nutrition care is a key component to achieve or maintain a healthy body weight, composition (i.e. lean and fat mass), and prevent chronic diseases after active cancer treatment [11]. Several clinical guidelines and position statements highlight the importance of nutrition interventions during and after cancer treatment to prevent malnutrition [7, 11–13]. However, the access to nutrition care and where nutrition information is sourced after active treatment and into the survivorship phase of cancer care is less well known.

People with cancer and their carers, across several cancer types, are highly motivated to seek out nutrition information in an attempt to gain control over their health, improve their quality of life, and reduce the risk of cancer recurrence [14–17]. Access to health care in Australia (including nutrition services) is universal and is considered amongst the best in the world in both public and private sectors. The nutrition needs for people with cancer are highly variable and depend on many clinical factors (i.e. cancer type and location, treatment type, and prevalence of side effects), as such not all people diagnosed and treated with cancer require access to a dietitian and/or individualised nutrition counselling. The majority of nutrition services within Australian hospitals conduct routine malnutrition risk screening and subsequently prioritise “at risk” cancer patients in order to optimise treatment outcomes, reduce the length of stay in hospital, and improve quality of life and survivorship [7]. Conversely for cancer patients at “low risk” of malnutrition (i.e. breast and prostate cancer), access to dietitian services can be limited and nutrition may not be routinely discussed in the clinical setting, despite high interest in nutrition [18]. Access to outpatient nutrition services after active cancer treatment varies across organisations and community-based services and private practice nutrition services usually come at an out-of-pocket cost. Recent reviews have highlighted that after the completion of active treatment (i.e. surgery, chemotherapy, radiotherapy) across several cancer types, that people often turn to the internet and other forms of digital media for nutrition information to improve health and wellbeing [19, 20]. With the increased volume of nutrition-related information readily available on the internet, it is important to understand both the patient and carer perceptions of publicly available nutrition information after completing active cancer treatment.

Research to date has focused on experiences with nutrition information and unmet dietary needs in people with cancer undergoing treatment. However, less is known about the availability and accessibility of nutrition services or information after cancer treatment from the patient and carer. In addition, carers face several challenges in supporting patients, including adequate nutrition, after the completion of active cancer treatment [21, 22]. An understanding of the preferences, barriers, and where nutrition information and services are sourced will help inform the design of appropriate nutrition services and resources to meet patient and carer needs after treatment. This study aimed to explore the accessibility of cancer-related nutrition services and information, whether available resources meet the patient and carer nutritional needs and understand the preferences for and barriers to accessing nutrition information after cancer treatment.

## Methods

This study utilised a mixed method design of a cross-sectional survey and semi-structured interviews to collect both quantitative and qualitative data from people with cancer and carers regarding their experience in accessing nutrition services and information after treatment. Ethical approval was received from the Human Ethics Advisory Group (HEAG), Faculty of Health at Deakin University on the 4th of August 2021, approval number HEAG-H 102_2021.

### Survey development

A 17-item survey was designed using Qualtrics (Qualtrics Provo, UT). The survey was tested for face validity by the research team members, two allied health practitioners (external to the research team), and two randomly selected adults receiving outpatient cancer care at Peter MacCallum Cancer Centre. Two patients and two allied health practitioners were approached by a member of the research team to complete face validation, which included written feedback on the readability and appropriateness of Likert scale questions and responses provided. The questionnaire was trialled across multiple devices. From consumer feedback, the question order, list of responses, structure, and wording of questions was refined before dissemination online (Supplementary Table 1). The survey focused on three key areas: (1) availability of nutrition services, (2) nutrition information searched after cancer treatment, and (3) barriers and preferences for nutrition information after cancer treatment. The survey also captured participant demographics including age, gender, postcode, cancer type, and treatment. A range of 5-point Likert scales was used to assess satisfaction with nutrition advice received (1 = not satisfied, 2 = slightly satisfied, 3 = moderately satisfied, 4 = very satisfied, 5 = extremely satisfied), level of agreement with various aspects of the questionnaire (1 = strongly disagree, 2 = disagree, 3 = slightly agree, 4 = agree, 5 = strongly agree), or the usefulness of nutrition information searched (1 = not useful at all, 2 = slightly useful, 3 = moderately useful, 4 = very useful, 5 = extremely useful).

### Participants and setting

The survey was open to adults who had finished cancer treatment or their carers between August 2021 and March 2022. Eligibility criteria included (1) adults (≥ 18 years) with a diagnosis of cancer, (2) who had finished active cancer treatment or care for an adult with cancer who had finished treatment, (3) had access to a smartphone and internet, and (4) were able to understand English. Participation was voluntary and consent was obtained by selecting “I agree to the above eligibility” to have their responses used for research purposes in Qualtrics (Qualtrics, Provo, UT) prior to starting the survey.

### Survey distribution and recruitment

The survey was distributed through several cancer-specific organisations. The organisations include Bowel Cancer Australia, Deakin Institute for Physical Activity and Nutrition (IPAN), Peter MacCallum Cancer Centre, The Cancer Council (Victoria, Tasmania, Western Australia), Breast Cancer Network Australia, Carers Couch, Counterpart and North Western Melbourne Primary Health Network (NWMPHN), People Bank, and the Prostate Cancer Foundation Australia (PCFA). The above organisations distributed information about the study and a link to the Qualtrics survey via email invitation, e-newsletters, or social media platforms to relevant consumer networks. Dissemination of the survey to potential participants was blinded from the investigators and anonymous.

### Semi-structured interviews

A sub-group of participants was recruited on a rolling basis to partake in a semi-structured interview exploring participant experiences with nutrition services and information after active cancer treatment. After completing the Qualtrics survey, a link was provided to a separate database for participants to register their interest in participating in an interview. Contact was made with interested participants to explain the interview process and a mutually convenient time was agreed. Interviews took place over Zoom Video Communications Inc. 2016 and participants provided verbal consent. An interview guide scripted the introduction and interview questions (Supplementary material 2). The audio of each interview was recorded using Otter.Ai (available at https://otter.ai/). Two members of the research team conducted the interviews. To evaluate the interview guide, B.J.B interviewed two participants and observed S.D interview two participants prior to S.D leading the remaining interviews.

### Statistical analysis

Statistical analysis was performed in *R* (Version 3). Participant characteristics were summarised using descriptive statistics. Likert scale questions were plotted in the *likert* R package (Bryer, J., Speerschneider, K.). Cancer types were recoded as high or low nutrition risk based on prevalence of malnutrition. High-risk cancer types included haematology, head and neck, gastrointestinal, lung, and other (palliative). Lower-risk cancer types included breast, genitourinary, gynaecology, and skin. Participants with a lower-risk primary cancer (i.e. breast and prostate) and high-risk secondary cancer (i.e. head and neck) were classified as high risk [5, 23]. Participant postcodes were classified as either rural or urban based on the Australian Bureau of Statistics Statistical Geography Standard [24]. Responses to Likert scales for agreeance and satisfaction were grouped into binomial categories due to the small sample size in this study to adequately compare between Likert scale responses. Agreeance Likert scales were collapsed to: agree = “strongly agree”, “agree”, “slightly agree” and disagree = “strongly disagree”, “disagree”. The same level of grouping was applied to satisfaction responses. Usefulness Likert scales were collapsed to either very-extremely useful = “very useful” plus “extremely useful”, slightly-moderately useful = “slightly useful” plus “moderately useful”, and non-useful = “not at all useful”.

Transcribed interviews were cleaned and exported to NVivo qualitative data analysis software; QSR International Pty Ltd. Version 12, 2018. All identifying details were removed before data cleaning. Analysis was guided by thematic analysis, consisting of single-line coding of interview responses, identifying key concept from the data, to thematically analyse the responses to broader concepts. Themes were not predefined but led from the coding of participant response [25]. All interviews were coded independently by S.D and B.J.B. The codes were collated by B.J.B who led the thematical analysis. Themes were discussed with S.D for agreeance. Themes are presented with quotes in the results.

## Results

A total of 149 participants completed the survey, with 139 being adults with cancer and ten carers of adults with cancer (Table 1). The majority of participants were male (69%) and had one diagnosis of cancer (91%). The most common cancer diagnosis was prostate (63%), and 77% of all participants were treated with surgery. Thirteen participants had two or more secondary cancers. Most participants resided in an urban location (70%) and were classified as being at low nutrition risk (71%).

**Table 1:** Summary of participant characteristics

Characteristics	Survey			Interview^a^
	All	Cancer patients	Carers	
* N*	149 (100%)	139 (93%)	10 (7%)	14 (100%)
Age (range)	63.4 (32–83)	64.5 (32–83)	54.1 (32–75)	65.5 (54–77)
Gender				
Female	47 (31%)	37 (27%)	10 (100%)	10 (71%)
Male	102 (69%)	102 (73%)	0 (0%)	4 (29%)
Place of residence^b^				
Victoria	58 (39%)	55 (40%)	3 (30%)	-
Western Australia	27 (18%)	26 (19%)	3 (30%)	-
Queensland	24 (16%)	21 (15%)	1 (10%)	-
New South Wales	23 (16%)	20 (14%)	3 (30%)	-
Tasmania	12 (8%)	12 (9%)	0 (0%)	-
South Australia	4 (3%)	4 (3%)	0 (0%)	-
Geographic location^b^				
Urban	103 (70%)	98 (71%)	5 (50%)	-
Rural	45 (30%)	40 (29%)	5 (50%)	-
Diagnosis				
Single cancer diagnosis	136 (91%)			
Prostate, testicular, penis	85 (57%)	78 (56%)	7 (70%)	4 (28%)
Breast	14 (9%)	14 (10%)	0 (0%)	3 (21%)
Head and neck	14 (9%)	13 (9%)	1 (10%)	1 (7%)
Gastrointestinal	9 (6%)	8 (6%)	1 (10%)	5 (36%)
Cervical/ovarian	6 (4%)	6 (4%)	0 (10%)	1 (7%)
Other	6 (4%)	5 (4%)	1 (0%)	-
Blood	2 (1%)	2 (1%)	0 (0%)	-
Treatments received for primary cancer				
Surgery	113 (77%)	107 (77%)	6 (60%)	13 (92%)
Radiotherapy	65 (44%)	58 (42%)	7 (70%	6 (43%)
Chemotherapy	44 (30%)	38 (27%)	6 (60%)	5 (36%)
Hormone therapy	40 (27%)	35 (25%)	5 (50%)	-
Other	13 (9%)	12 (9%)	1 (10%)	-
Immunotherapy	6 (4%)	6 (4%)	0 (0%)	-

^a^All participants with a cancer diagnosis

^b^Data available for *n* = 148

### Survey results

Overall, 35 (23%) participants indicated that they received nutrition information after cancer treatment from a dietitian, whilst 20 (14%) received nutrition information from a different health care professional, and 89 (62%) did not receive nutrition information from any health care professional post treatment. Of the participants that received nutrition information from any health care professional, 24 (68%) were diagnosed with a cancer considered high nutrition risk. Collectively, participants reported that the nutrition information received was understandable (92%, *n* = 48), beneficial (90%, *n* = 47), and improved general health (77%, *n* = 40).

 Approximately half of the participants (55%) actively searched for cancer nutrition information as shown in Table 2. Participants searched multiple (median 2; range 1–8) sources to access cancer nutrition information. Overall, 62% of participants that searched the internet for cancer nutrition information and the internet remained the highest-ranked source of information when nutrition risk (low = 58%; high = 71%) and geographic location (rural = 76%; urban = 57%) were compared. Participants indicated that the nutrition information was easy to understand (89%), beneficial to their needs (87%), specific to their treatment (84%), and resulted in a change to diet (79%) whilst approximately half (52%) indicated the information was conflicting.

**Table 2:** Participants perception of cancer nutrition information

		Nutrition risk		Geographic location	
	All	Low	High	Rural	Urban
Sources of nutrition information searched ranked by frequency *n* (%)	83 (55%)	55 (66%)	28 (33%)	26 (32%)	56 (68%)
Internet	1 (62%)	1 (58%)	1 (71%)	1 (76%)	1 (57%)
Booklet/pamphlet/factsheet	2 (52%)	2 (50%)	2 (53%)	2 (57%)	2 (50%)
Cancer Council Australia	3 (34%)	3 (29%)	3 (46%)	3 (50%)	3 (28%)
Support group	4 (33%)	5 (23%)	2 (53%)	2 (57%)	5 (23%)
Specific Cancer Organisations	5 (31%)	4 (27%)	4 (39%)	4 (42%)	4 (44%)
Relevance of nutrition information^a^ *n* (%)	78 (52%)	53 (68%)	25 (32%)	25 (32%)	52 (67%)
Was specific to my treatment	66 (84%)	43 (81%)	23 (92%)	22 (88%)	43 (77%)
Was specific to my cancer	60 (77%)	37 (70%)	23 (92%)	18 (72%)	41 (73%)
Was personalised/individualised to my condition	46 (59%)	29 (54%)	17 (68%)	19 (76%)	26 (46%)
Was beneficial to my needs	68 (87%)	46 (86%)	22 (88%)	24 (96%)	43 (77%)
Was easy to understand	70 (89%)	46 (86%)	24 (96%)	23 (92%)	46 (82%)
Was easy to find	57 (73%)	41 (77%)	16 (64%)	21 (84%)	35 (62%)
Provided conflicting information	41 (52%)	26 (49%)	15 (60%)	13 (52%)	27 (48%)
Was practical for me	65 (83%)	43 (81%)	22 (88%)	23 (92%)	41 (73%)
Resulted in me changing my diet	62 (79%)	41 (77%)	21 (84%)	23 (92%)	38 (67%)

^a^The proportion reporting satisfied

**Fig. 1:**
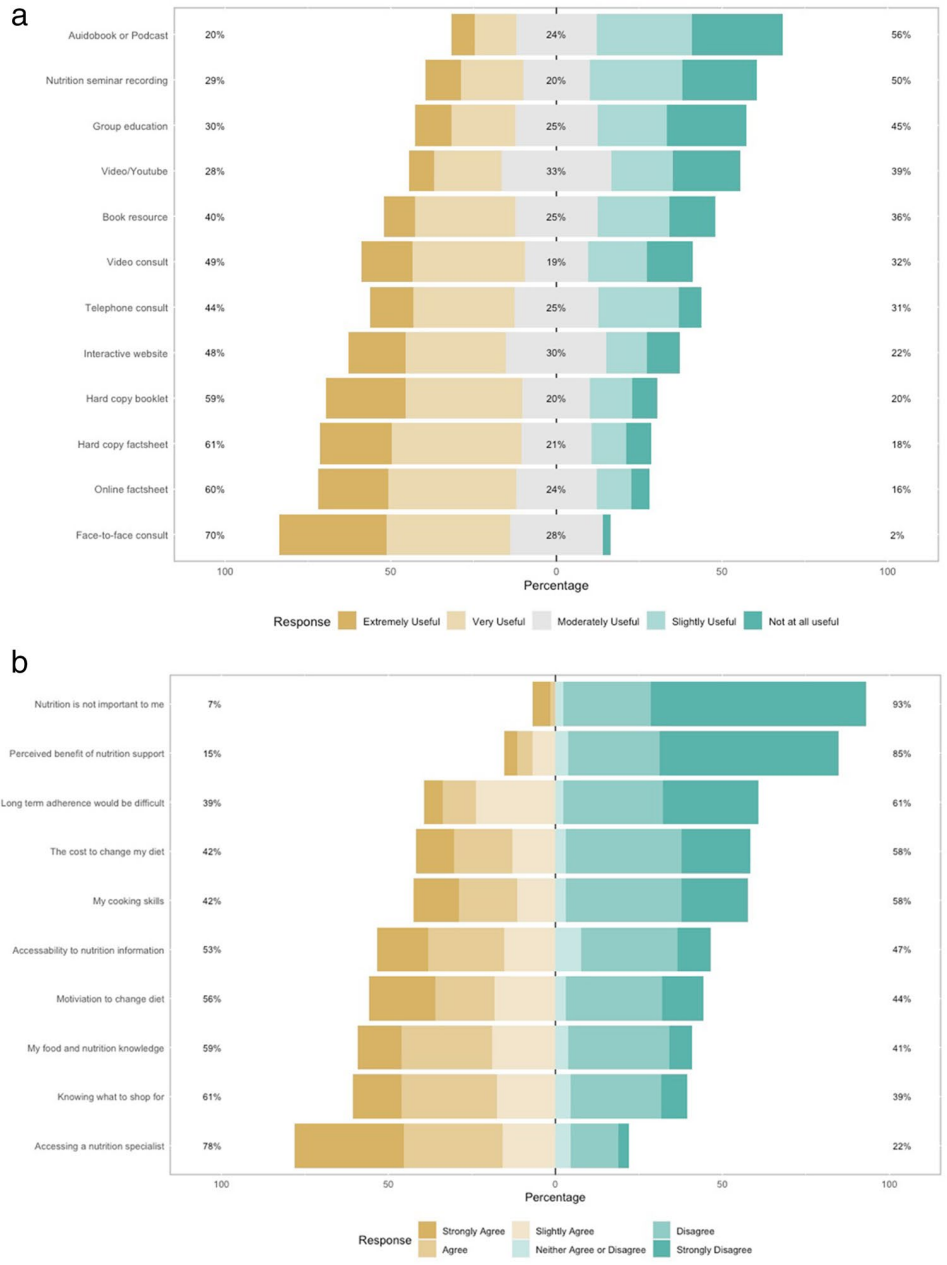
**a** Preferences for accessing nutrition information after cancer treatment. **b** Barriers for accessing nutrition information after cancer treatment

A majority of participants (70%, indicated very-extremely useful) stated face-to-face consultations with a nutrition specialist is the preferred modality to receive nutrition information (Fig. 1a). Hard copy factsheets were the second highest preference with 61% indicating this modality of nutrition care would be “very-extremely useful”. Participants residing in a rural-classified postcode indicated face-to-face consultations are “very-extremely useful” (*n* = 25; 65%) or “slightly-moderately useful” (*n* = 14; 36%). Whilst participants with a high nutrition risk after cancer diagnosis reported face-to-face consultations as the preferred modality of nutrition information (*n* = 28; 77%) reporting very-extremely useful). Proportions for other modes of nutrition information showed no clear preference across nutrition risk or geographic location. Conversely, most participants indicated accessing a nutrition specialist (78%) is the first ranked barrier for nutrition after cancer treatment (Fig. 1b). Participants also indicated that “knowing what to shop for” and their “food and nutrition knowledge” were also key barriers to implementing nutrition advice after treatment.

### Semi-structured interviews

Sixteen participants registered interest, two failed to attend, and after data saturation, a total of fourteen participants completed the semi-structured interviews. Table 1 demonstrates that interview participants had a variety of cancer types and treatments. Three major themes were derived from the data including (1) preferred referral and timing of nutrition services, (2) lack of confidence in publicly available nutrition information, and (3) streamlining nutrition services for greater access.

### Theme 1: preferred referral and timing of nutrition services

Participants revealed that access to nutrition services after cancer treatment is scarce. Most notably, participants reported that they feel nutrition care is important for managing ongoing symptoms after cancer treatment (i.e. weight and muscle loss), or to reduce the risk of cancer recurrence. Most participants reported feeling isolated after treatment and did not know where to look for correct nutrition information or to engage with nutrition specialists. For example: *“The only way I found out about the [nutrition service] was that there was a flyer in the oncologist’s rooms. So, the onus was on me… Who knows how I would have found that kind of help [P4].”* Whilst participants noted the importance of nutrition after treatment, not all experiences with a nutrition professional were positive: *“So I did see a dietitian, but it was such a disappointing experience. They literally sat in front of a computer and printed out material from the internet and handed that to me, and I just left there thinking, oh my God, I could have done that [P11].”* Most participants indicated they would have liked nutrition care after treatment from a health professional where they received treatment: *“I think part of your discharge from treatment should be the whole gamut. I feel that it should be a physiotherapist, a social worker, if you're struggling with coping with what you've gone through, but I also feel a dietitian should be in every treatment team… it’s that important [P12].”*

Most participants discussed when nutrition care should be provided to them after cancer treatment. Participants clearly indicated that they would prefer nutrition care from a dietitian post-treatment, and several participants noted the days leading up to a hospital discharge is when they would ideally prefer a suite of nutrition information (i.e. a consultation, nutrition handout/information and how to access community services). Some suggested the acute post-treatment phase can provide motivation for dietary change: *“It's very difficult to change a [dietary] pattern once you've gone back into your old routine. So, I think the earlier you got that [referring to nutrition], you got that knowledge and that information, the better [P10].”* Others strongly believe nutrition services should be integrated as part of standard care once treatment has finished for long-term maintenance of health. For example: *“I think the oncologist or the cancer surgeon, needs to have it as part of their checklist at the six or three-monthly checkups, and refer on if needed [P14].”*

### Theme 2: lack of confidence in publicly available nutrition information

Participants used multiple sources to engage with cancer nutrition information including but not limited to, books/pamphlets, support groups, and the internet. The internet has provided a wide-reaching means for nutrition information; however, knowing where to find credible evidence-based nutrition information was a barrier to many participants. Nutrition information on the internet appears to be contradictory and a point of confusion to participants. For example: *“I know there's a massive amount of stuff on the web. But sorting out what's worth reading from what isn't, is not always easy [P5].”* Participants indicated that they need to trust the source providing nutrition information. Government organisations or cancer-specific foundations were noted as trustworthy sources: *“[organisation] has been great because they feel like I can really trust them to provide information that is evidence-based and that I can kind of rely on [P12].”* Beyond trusting the source, participants indicated they needed to understand the research and how specific dietary components influence side effects or reduce cancer progression.

### Theme 3: streamlining nutrition services for greater access

Most participants indicated that having a conversation about nutrition with a member of the health care team is sufficient for them to ask questions and seek a referral if needed. Others suggested nutrition should be integrated into a broader lifestyle package of information for after cancer treatment. However, embedding evidence-based nutrition information to an online resource that is readily available and accessible was a common theme from participants. For example: *“I would wish somebody said here is a booklet or an excellent website that tells me, this is what you do need [referring to nutrient or food-group requirements], these are the things that will help you regain your strength and to stay healthy specific to cancer… I would like a site that has people talking about their experiences, that is visually informative, and wouldn’t be somebody's talking so much but showing what foods would be good [P2].”* Participants felt it was important to have a service that was centralised and easy to access when needed after cancer treatment. Telehealth was mentioned numerous times as a convenient option for participants to access evidence-based nutrition care that was personalised. For example: *“You could ring up and say that I'm struggling with whatever and there is somebody on the other end of the phone that can help you directly [P13].”*

Participants clearly indicated that the nutrition information must be provided by a nutrition specialist, such as a dietitian, with knowledge of cancer and how to manage treatment, side effects, and recurrence. For example: “*I think it probably holds more weight if the person providing [referring to nutrition] has that nutritional expertise and background. I think that's really the most important thing [P10].”* Reducing confusion and contradictory nutrition information is crucial for participants to engage in the service and result in an appropriate change of diet. Participants noted it was problematic when contradictory advice was provided between services: *“I put great faith in expert knowledge. Interestingly, back to that comment I made at the beginning about ‘dietitian one,’ their information contradicted a lot to ‘dietitian two’ and dietitian two was not surprised, they said that's not unusual [P4].”*

## Discussion

This study evaluated the access to nutrition care and information after active cancer treatment through a mixed-methods design and revealed four important findings: (1) nutrition is an important factor for people with cancer and carers after treatment, yet 23% of participants in this study received nutrition care from an accredited practising dietitian after treatment; (2) 78% of participants indicated that accessing a nutrition specialist is the main barrier to obtaining adequate nutrition information after treatment; (3) people with cancer would prefer nutrition information that focused on preparing for after treatment concerns as early as possible; and (4) most utilised the internet to engage with nutrition information yet reported the information was conflicting and lacked confidence in the credibility of publicly available information on this topic. Our findings are in accordance with previous reviews that highlight referrals to nutrition services after cancer treatment are scarce [19, 20], and further work is required by health services treating people with cancer and community-based nutrition services to enable improved access to appropriate nutrition care post cancer treatment.

The present study builds upon previous work reporting that 32% of people with cancer and carers received a consultation with a dietitian during treatment and many felt unsupported with nutrition [26]. This study further indicates that people with cancer and carers continue to be motivated and interested in accessing nutrition services after active cancer treatment but continue to experience barriers in referrals with only 23% consulting with a dietitian for post-treatment nutrition advice. This is particularly concerning for people with cancer at high nutrition risk where evidence-based nutrition care and information should be readily accessible and available. Several barriers inhibit people with cancer from accessing nutrition services after cancer treatment, which include, but are not limited to a lack of knowledge regarding effective referrals, transitions and pathways of post-treatment nutrition care across health settings, limited established pathways of care to community-facing nutrition services, and the cost of private services [19]. However, there is emerging evidence regarding models of care to support improved coordination and strengthen referral pathways across acute and community-based settings for people with cancer [27]. Novel models of survivorship care that are designed for more effective, sustainable and patient-centred survivorship care (i.e. stratified risk, shared care) provide a possible way forward to improve access to nutrition services after active cancer treatment [28]. Telehealth may also provide a feasible option to facilitate post-treatment nutrition care for people with cancer in remote and rural locations [29]. Telehealth nutrition interventions have improved multiple cancer-related outcomes in breast [30] and more recently head and neck cancer [31]; however, telehealth might not be the preferred modality of nutrition care for all people with cancer. Thus, further work is required to develop models of care that triage people with cancer for ongoing nutrition care where appropriate, to hospital, community and/or shared care.

The present study highlighted the importance of when dietary information is delivered. Interviews with participants revealed they would prefer nutrition be discussed as early as possible to prepare them for when treatment ceases. Similarly, previous studies report there is a need for health information (including nutrition) to be repeatedly discussed to account for changing needs and information overload [32]. Our findings revealed that there is a preference for nutrition to be discussed at post-treatment medical appointments and for referral to nutrition services where appropriate. Importantly, a previous Australian study highlighted that medical professionals understand the importance of screening for malnutrition and incorporating dietetics into the treatment plan of people with cancer [33]. As such, including the malnutrition screening tool [34], which is a two-question tool about dietary intake and weight status, would help identify patients that require a referral for nutrition assessment and intervention post-treatment.

People with cancer and carers in our study indicate that they actively searched for nutrition information after treatment, predominately through the internet, to inform their dietary habits. These findings are consistent with a growing body of evidence that suggests up to 80% of people with cancer seek nutrition information through the internet [35]. Furthermore, most participants in our study indicated that the nutrition information was conflicting and consequently may lead to dietary changes that are not aligned with best-practice evidence [19]. Our findings support previous surveys and qualitative interviews that revealed people with cancer are potentially vulnerable to misinformation on nutrition [23] and are left questioning the quality of the evidence [36]. Recent investigations revealed that level of education influences the ability to locate and interpret health-related information (including nutrition) in healthy adults [37]. Given the complexity of nutrition issues and concerns that arise during and after cancer treatment, health literacy is an important skill needed to decipher the volume of non-evidence-based health information in cancer [38]. Emerging research indicates nutrition literacy is a wider concern for adults with a chronic disease [39], or women with breast cancer [40]. Whilst nutrition literacy is relatively unknown across other cancers, this may explain why barriers such as “knowing what to shop for” and “my food and nutrition knowledge” were prominently listed as barriers to accessing nutrition information in this study.

This mixed methods study evaluated access to nutrition services and information for people and carers post cancer treatment. Whilst this study is beneficial in identifying gaps in nutrition information and services after cancer treatment within Australia, it does have some notable limitations. The small sample size, with the majority of our sample being diagnosed with prostate cancer, likely skewed our results and are not proportional to all people with cancer post treatment. People at risk, or diagnosed with malnutrition (i.e. head and neck or gastrointestinal cancers), require individualised dietetic assessment and management during cancer treatment, yet whether dietetic services and evidence-based nutrition information are readily available after active treatment varies between settings and requires further investigation. The heterogeneous sample in the semi-structured interviews is limited to evaluating the broader issues in accessing nutrition services and information across different cancer types. Future studies that target single tumour types or treatments which predispose patients to malnutrition (i.e. head and neck or lung cancer) would likely provide unique insight to whether patients are being consulted by a dietitian as an outpatient, have moved to the community or private sector, or are not being seen and accessing information online. Carers in our study were underrepresented and more research is required to understand their needs and experiences with nutrition information after treatment. Given our study also found the internet was a barrier to accessing nutrition information, the fact that our survey was only available online may have excluded the experiences of people with cancer and carers without access to or who are less proficient with the internet. Comparisons between participant characteristics such as nutrition risk (i.e. low or high risk of malnutrition) or geographical location (i.e. rural and regional) and survey responses were not feasible given most participants in our sample were at low nutrition risk and lived in an urban region. This study was specific to people and carers who have completed active cancer treatment in Australia and limits comparisons to other countries with different health care systems. Lastly, it is expected that participants interested in nutrition were more likely to complete this survey which may also contribute to skewed data.

## Conclusion

This study identified that many people with cancer and carers experience barriers to accessing nutrition care and information after active cancer treatment in Australia. Most participants in this study turned to the internet to access nutrition information; however, the information was conflicting, and participants lacked confidence in the information available online. Ensuring nutrition is discussed by medical staff as early as possible in the cancer care continuum and also through to post-treatment appointments provides people with cancer the opportunity for referral to an accredited practising dietitian for assessment and management. Future work must evaluate different models of care that facilitate access to evidence-based nutrition care and information after treatment.

### Supplementary Information

Below is the link to the electronic supplementary material.

Supplementary file1 (DOCX 40 KB)

Supplementary file2 (DOCX 27 KB)

## Data Availability

De-identified data from only the survey will be made available upon request following appropriate data sharing agreements.
