# Supplementary material for: Access to nutrition services and information after active cancer treatment: a mixed methods study
Source: J Cancer Surviv. 2023 Feb 23;18(1):176–85. doi: 10.1007/s11764-023-01352-x (PMC10866769; doi:10.1007/s11764-023-01352-x)
Supplement: Supplementary file 1 — Supplementary file1 (DOCX 40 KB) [file 11764_2023_1352_MOESM1_ESM.docx]

**Appendix A:**

**Survey Questions**

*Demographic questions:*

1. Are you a patient or a carer?
2. Age ______
3. Gender

☐ Male

☐ Female

☐ Prefer not to answer

1. Postcode __________
2. Which of the below best describes your (or the person you care/cared for) cancer diagnosis?

☐ Lung

☐ Breast

☐ Prostate, testicular, penis

☐ Head and neck (mouth, nose, throat, voice box)

☐ Skin

☐ Brain and spine

☐ Gastrointestinal (upper) – specify type gastrointestinal cancer: ________________

☐ Gastrointestinal (lower) – specify type gastrointestinal cancer:  ________________

☐ Blood

☐ Bladder/kidney

☐ Cervical/ovarian

☐ Bone or muscle/nerves (sarcoma)

☐ Unknown primary

☐Other, please specify cancer diagnosis: _______________________

1. Which cancer treatment(s) did you receive? (or for the person you care/cared for; Select all that apply)

☐ Surgery

☐ Chemotherapy

☐ Oral

☐ Intravenous (drip)

☐ Radiotherapy

☐ Stem cell transplant

☐ Immunotherapy

☐ Hormone therapy

☐ Other, please specify cancer treatment:_______________________

1. Were you provided with any nutrition information that was focused on nutrition and/or diet for **when/after treatment finishes** from the following list of health professionals?

| **Health professionals** | **For every health professional selected, how satisfied were you with the nutrition information** |
| --- | --- |
| ☐ Dietitian at my hospital | ☐ Not satisfied |
| ☐ Community dietitian | ☐ Slightly satisfied |
| ☐ Private practice dietitian | ☐ Moderately satisfied |
| ☐ Nurse at my hospital | ☐ Very satisfied |
| ☐ Doctor at my hospital | ☐ Extremely satisfied |
| ☐ My GP |  |
| ☐ Other __________ |  |
| ☐I did not remember receiving nutrition information from a health professional | Move to Q10 |

1. The nutrition information I received for **when/after treatment finishes** focused on ____ (Tick all that are relevant to you)

| **Nutrition advice** | **If ticked, when was this nutrition information provided to you** |
| --- | --- |
| ☐ Healthy eating | ☐ Before treatment |
| ☐ Treatment side effect: nausea | ☐ During treatment |
| ☐ Treatment side effect: vomiting | ☐ At the end of treatment |
| ☐ Treatment side effect: loss of appetite | ☐ < 3 months after treatment |
| ☐ Treatment side effect: taste changes | ☐ 3-6 months after treatment |
| ☐ Treatment side effect: dry mouth | ☐ > 6 months after treatment |
| ☐ Treatment side effect: mouth ulcers |  |
| ☐ Treatment side effect: constipation |  |
| ☐ Treatment side effect: diarrhoea |  |
| ☐ Losing body weight |  |
| ☐ Maintaining my body weight |  |
| ☐ Gaining body weight |  |
| ☐ Maintaining my muscle mass |  |
| ☐ Regaining my muscle mass |  |
| ☐ Included oral nutrition supplements (i.e. Sustagen) |  |
| ☐ Cancer recurrence |  |
| ☐ Other __________ |  |

1. The nutrition information I received from a health professional for **when/after treatment finishes** _______________

|  | Strongly disagree | Disagree | Slightly agree | Agree | Strongly Agree |
| --- | --- | --- | --- | --- | --- |
| Was personalised to me | ☐ | ☐ | ☐ | ☐ | ☐ |
| Was beneficial to my needs | ☐ | ☐ | ☐ | ☐ | ☐ |
| Was easy to understand | ☐ | ☐ | ☐ | ☐ | ☐ |
| Resulted in me changing my diet | ☐ | ☐ | ☐ | ☐ | ☐ |
| Improved my general health | ☐ | ☐ | ☐ | ☐ | ☐ |
| Improved my physical health | ☐ | ☐ | ☐ | ☐ | ☐ |
| Improved my mental health | ☐ | ☐ | ☐ | ☐ | ☐ |
| Helped me to recover from cancer treatment | ☐ | ☐ | ☐ | ☐ | ☐ |

1. I actively searched for nutrition information that was focused on nutrition and/or diet **after treatment** from the list of options below?

|  | If ticked, can you provide details of what you searched/read |
| --- | --- |
| ☐ Cancer council |  |
| ☐ Specific council/organisations to my cancer (i.e prostate cancer foundation) |  |
| ☐ Internet |  |
| ☐ Government website/documents |  |
| ☐ Blog |  |
| ☐ Books |  |
| ☐ Booklets/pamphlets |  |
| ☐ Fact sheets |  |
| ☐ Hospital website |  |
| ☐ Cancer support groups |  |
| ☐ Audiobook/podcasts |  |
| ☐ Video/Youtube |  |
| ☐ Journal articles |  |
| ☐ Other |  |
| ☐ I did not search for nutrition information | Move to Q12 |

1. The nutrition information I searched for after treatment ___________

|  | Strongly disagree | Disagree | Slightly agree | Agree | Strongly Agree |
| --- | --- | --- | --- | --- | --- |
| Was specific to my treatment | ☐ | ☐ | ☐ | ☐ | ☐ |
| Was specific to my cancer | ☐ | ☐ | ☐ | ☐ | ☐ |
| Was personalised/individualised to my condition | ☐ | ☐ | ☐ | ☐ | ☐ |
| Was beneficial to my needs | ☐ | ☐ | ☐ | ☐ | ☐ |
| Was easy to understand | ☐ | ☐ | ☐ | ☐ | ☐ |
| Was easy to find | ☐ | ☐ | ☐ | ☐ | ☐ |
| Provided conflicting information | ☐ | ☐ | ☐ | ☐ | ☐ |
| Was practical for me | ☐ | ☐ | ☐ | ☐ | ☐ |
| Resulted in me changing my diet | ☐ | ☐ | ☐ | ☐ | ☐ |

1. What best describes the changes to your diet since you have finished treatment?

|  | If yes, can you provide details of your diet change |
| --- | --- |
| ☐ Included more high energy high protein foods |  |
| ☐ Have started oral nutrition supplements (i.e. Sustagen) |  |
| ☐ I eat more plant based foods |  |
| ☐ I have removed foods |  |
| ☐ I have added foods |  |
| ☐ I now follow a specific diet | If selected, what diet are you following: |
|  | ☐ I now follow a Mediterranean diet |
|  | ☐ I now follow a Ketogenic diet |
|  | ☐ I now follow a Vegetarian diet |
|  | ☐ I now follow a Paleo diet |
|  | ☐ I now follow a Weight Watchers diet |
|  | ☐ Other: |
| ☐ Other: |  |
| ☐ I haven’t made any dietary changes | Move to Q13 |

1. What format of nutrition information for **when/after treatment finishes** would best suit your needs?

|  | Not at all useful | Slightly useful | Moderately useful | Very useful | Extremely useful |
| --- | --- | --- | --- | --- | --- |
| Hard copy fact sheets | ☐ | ☐ | ☐ | ☐ | ☐ |
| Hard copy detailed booklet | ☐ | ☐ | ☐ | ☐ | ☐ |
| Online fact sheet | ☐ | ☐ | ☐ | ☐ | ☐ |
| Interactive website | ☐ | ☐ | ☐ | ☐ | ☐ |
| Books | ☐ | ☐ | ☐ | ☐ | ☐ |
| Audiobook/podcasts | ☐ | ☐ | ☐ | ☐ | ☐ |
| Video/Youtube | ☐ | ☐ | ☐ | ☐ | ☐ |
| Consult with a health professional face-to-face | ☐ | ☐ | ☐ | ☐ | ☐ |
| Consult with a health professional via telephone | ☐ | ☐ | ☐ | ☐ | ☐ |
| Consult with a health professional via video call | ☐ | ☐ | ☐ | ☐ | ☐ |
| Group seminar/education | ☐ | ☐ | ☐ | ☐ | ☐ |
| Nutrition seminar recording | ☐ | ☐ | ☐ | ☐ | ☐ |
| Other | ☐ | ☐ | ☐ | ☐ | ☐ |

1. What health professional would you prefer to receive nutrition information from that was focused on **when/after treatment finishes**?

|  | If ticked, why would you prefer advice from this health professional |
| --- | --- |
| ☐ Dietitian at my hospital |  |
| ☐ Community dietitian |  |
| ☐ Private practice dietitian |  |
| ☐ Nurse at my hospital |  |
| ☐ General practice nurse |  |
| ☐ Doctor at my hospital |  |
| ☐ My GP |  |
| ☐ Other nutrition specialists __________ |  |
| ☐ Other _____ |  |

1. I believe nutrition information focused on **when/after treatment finishes** should _______

|  | Strongly disagree | Disagree | Slightly agree | Agree | Strongly Agree |
| --- | --- | --- | --- | --- | --- |
| Be general nutrition advice | ☐ | ☐ | ☐ | ☐ | ☐ |
| Be individual nutrition advice specific to me | ☐ | ☐ | ☐ | ☐ | ☐ |
| Include a meal plan | ☐ | ☐ | ☐ | ☐ | ☐ |
| Include advice on my treatment side effects | ☐ | ☐ | ☐ | ☐ | ☐ |
| Include recipes and education | ☐ | ☐ | ☐ | ☐ | ☐ |
| Include fact sheets | ☐ | ☐ | ☐ | ☐ | ☐ |
| Be focused on cancer recurrence | ☐ | ☐ | ☐ | ☐ | ☐ |
| Other | ☐ Please provide details on what nutrition information should be included | | | | |

1. What barriers do you feel make it challenging to receiving the right nutrition information for **when/after treatment finishes**?

|  | Strongly disagree | Disagree | Slightly agree | Agree | Strongly Agree |
| --- | --- | --- | --- | --- | --- |
| Accessing a nutrition specialist | ☐ | ☐ | ☐ | ☐ | ☐ |
| Accessibility to nutrition information (i.e. internet) | ☐ | ☐ | ☐ | ☐ | ☐ |
| My food and nutrition knowledge | ☐ | ☐ | ☐ | ☐ | ☐ |
| Knowing what to shop for | ☐ | ☐ | ☐ | ☐ | ☐ |
| My cooking skills and preparation of meals | ☐ | ☐ | ☐ | ☐ | ☐ |
| Confusion with different nutrition advice | ☐ | ☐ | ☐ | ☐ | ☐ |
| Costs to change my diet | ☐ | ☐ | ☐ | ☐ | ☐ |
| Motivation to change diet | ☐ | ☐ | ☐ | ☐ | ☐ |
| I don’t see any associated benefits with nutrition | ☐ | ☐ | ☐ | ☐ | ☐ |
| Nutrition is not important to me | ☐ | ☐ | ☐ | ☐ | ☐ |
| Long term adherence would be difficult | ☐ | ☐ | ☐ | ☐ | ☐ |
| Other: ______ |  |  |  |  |  |

1. Are there any other barriers to you access nutrition information (that are not covered above)? ________________
