# Supplementary material for: Access to nutrition services and information after active cancer treatment: a mixed methods study
Source: J Cancer Surviv. 2023 Feb 23;18(1):176–85. doi: 10.1007/s11764-023-01352-x (PMC10866769; doi:10.1007/s11764-023-01352-x)
Supplement: Supplementary file 2 — Supplementary file2 (DOCX 27 KB) [file 11764_2023_1352_MOESM2_ESM.docx]

**Supplementary material 2: Interview questions for semi-structured interviews**

| 1. | Can you tell me about your experiences transitioning from hospital to home?   - 1. Prompt: Side effects   2. Prompt: Did any occur after treatment |
| --- | --- |
| 2. | Have you noticed any issues with your weight or muscles since completing your cancer treatment?   1. Prompt: if poor historians: tighter clothes, increased/decreased belt size 2. Prompt: functional capacity |
| 3. | Can you tell me the impact COVID19 has had on nutrition/diet? |
| 4. | What, if any, changes have you made to your diet since completing your cancer treatment?   1. Prompt: related to side effects from treatment 2. Prompt: any drivers to making these changes? |
| 5. | Where have you searched for nutrition advice since completing your cancer treatment?   1. Prompt: blog, magazines, etc 2. Prompt: change diet from this advice |
| 6. | What, if any, nutrition advice have you been given since completing your cancer treatment?   1. Prompt: by who and what did this consist of, 2. Prompt: interested in nutrition advice? |
| 7. | What gives you confidence in nutrition advice/information?   1. Prompt: the health professional? 2. Prompt: evidence? |
| 8. | When would nutrition information for after/when treatment finishes suit you?   1. Prompt: And why |
| 9. | How could nutrition information be more accessible to you after hospital? |
